# Efficacy of ulinastatin for the treatment of patients with severe acute pancreatitis

**DOI:** 10.1097/MD.0000000000017644

**Published:** 2019-10-25

**Authors:** Jian-hui Yao, Wei-min Li

**Affiliations:** a Department of Critical Care Medicine; b Department of Emergency, Yulin No.1 Hospital, Yulin, China.

**Keywords:** efficacy, safety, severe acute pancreatitis, ulinastatin

## Abstract

**Background:**

The aim of this study is to explore the efficacy and safety of ulinastatin for the treatment of patients with severe acute pancreatitis (SAP).

**Methods:**

We will search randomized controlled trials which assess the efficacy and safety of ulinastatin for patients with SAP from the electronic databases of Cochrane Library, MEDILINE, EMBASE, CINAHL, PsycINFO, Scopus, CBM, Wangfang, VIP, and CNKI. All electronic databases will be searched from inception to the present with no limitations of language and publication status. Two researchers will carry out study selection, data extraction, and study quality assessment independently. Another researcher will help to resolve any disagreements between 2 researchers.

**Results:**

The outcomes include overall mortality, time of hospital stay, complications of systematic or local infection, multiple organ deficiency syndrome, health related quality of life (as measured as the 36-Item Short Form Health Survey), and adverse events related to nutrition.

**Conclusion:**

This study will provide evidence to evaluate the efficacy and safety of ulinastatin in the treatment of patients with SAP.

**Systematic review registration:**

PROSPERO CRD42019149566.

## 1 Introduction

Severe acute pancreatitis (SAP) is one of the most devastating diseases at the emergency clinical practice.^[1–3]^ It is characterized as an acute abdominal disease with a local pancreatic damage.^[4–6]^ It often quickly spreads into multiple organ dysfunction.^[7–8]^ Such condition is often associated with high morbidity and mortality.^[9–10]^ It has been estimated that its mortality rate still varies from 10% to 85% according to the various studies, although there is a decreasing tendency over the last decade.^[11–17]^ A variety of previous clinical studies have reported to use ulinastatin for the treatment of patients with SAP.^[18–23]^ However, no systematic review and meta-analysis has investigated the efficacy and safety of ulinastatin for the treatment of patients with SAP. Thus, this study will firstly assess the efficacy and safety of ulinastatin for treating SAP systematically.

## 2 Methods

### 2.1 Dissemination and ethics

This study is expected to be published at peer-reviewed journals. Ethical approval is not needed, because all data in this study has been published.

### 2.2 Study registration

This study protocol has been registered on PROSPERO CRD42019149566. We report the results of this study according to the Cochrane Handbook for Systematic Reviews of Interventions and the Preferred Reporting Items for Systematic Reviews and Meta-Analysis Protocol statement guidelines.^[24]^

### 2.3 Inclusion criteria for study selection

#### 2.3.1 Types of studies

We will include randomized controlled trials (RCTs) of ulinastatin for the treatment of patients with SAP without language limitation. However, non-clinical studies, case studies, observational studies, and non-RCTs will be excluded.

#### 2.3.2 Types of participants

Patients diagnosed with SAP will be considered for inclusion, while ethnicity, gender, and age are not limited.

#### 2.3.3 Types of interventions

The treatment group has utilized the ulinastatin without restrictions of dose and frequency.

The control group has received any interventions, such as placebo, except any forms of ulinastatin.

#### 2.3.4 Type of outcome measurements

The outcome measurements consist of overall mortality, time of hospital stay, complications of systematic or local infection, multiple organ deficiency syndrome, health related quality of life (as measured as the 36-Item Short Form Health Survey), and adverse events related to nutrition.

### 2.4 Search methods for the identification of studies

#### 2.4.1 Electronic database searches

Literature records will consist of electronic databases and manual searches. We will search Cochrane Library, MEDILINE, EMBASE, CINAHL, PsycINFO, Scopus, CBM, Wangfang, VIP, and CNKI. All electronic databases will be searched from inception to the present without restrictions of language and publication status. All RCTs of ulinastatin in the treatment of patients with SAP will be considered. The search strategy example for Cochrane Library is presented in Table 1. Similar search strategies of other electronic databases will also be used.

**Table 1:**
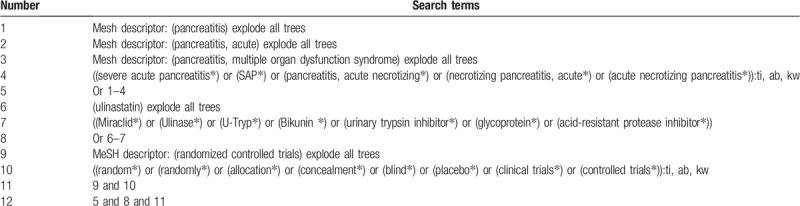
Search strategy for Cochrane Library database.

#### 2.4.2 Search for other resources

Aside from all electronic databases, dissertations, conference proceedings, ongoing studies, clinical registry, and reference list of included studies will be searched.

### 2.5 Data collection and analysis

#### 2.5.1 Study selection

**Figure 1:**
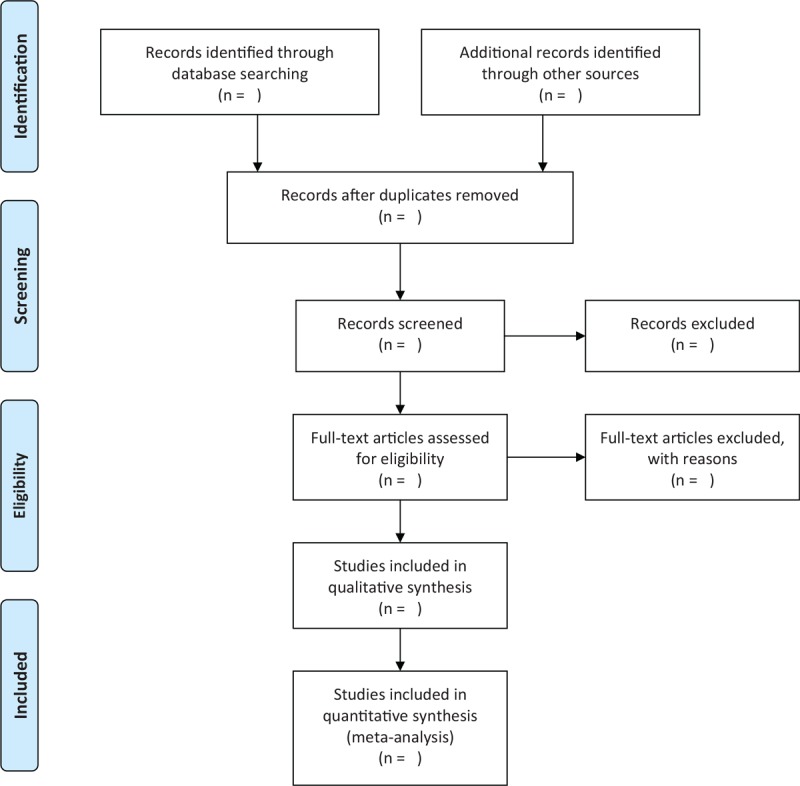
Flow diagram of study selection.

Two researchers will independently read the titles and abstracts of searched literature records to exclude irrelevant records. We will also read the full texts of remaining studies to determine whether those studies can be finally included. Any divergences regarding the study selection between 2 researchers will be solved by a third researcher. We will present the study selection process in the flow chart in Figure 1.

#### 2.5.2 Data collection and management

Two researchers will retrieve the following information independently according to the pre-designed data extraction sheet. If there is a disagreement, another researcher will be involved to solve such divergence. The extracted information include study details (title, first author, year of publication, et al), patient details (baseline characteristics, diagnostic criteria, inclusion and exclusion criteria, et al), study methods (sample size, randomization, blind, concealment, et al), treatment details (dose, frequency, duration, etc), and outcomes (outcome measurements, safety, etc et al).

#### 2.5.3 Risk of bias assessment

Two researchers will evaluate the methodological quality for each included studies using Cochrane Handbook for Systematic Reviews of Interventions tool. This tool covers 7 aspects, and each 1 is further classified into high, unclear or low risk of bias. Another research will help to resolve any disagreements between 2 researchers.

#### 2.5.4 Measurement of treatment effect

For enumeration data, we will record the risk ratio and 95% confidence intervals, while for continuous data, we will record the mean difference or standardized mean difference and 95% confidence intervals.

#### 2.5.5 Dealing with missing data

If there is missing or insufficient data, we will contact primary authors to require it. If it is not obtained, we will synthesize and analyze the available data, and will also discuss the possible impacts of the missing data.

#### 2.5.6 Assessment of heterogeneity

Heterogeneity among eligible studies will be identified by *I*^*2*^ test. When *I*^*2*^ ≤ 50%, heterogeneity is reasonable, while when *I*^*2*^ > 50%, the heterogeneity is considered as substantial.

#### 2.5.7 Data synthesis

We will utilize RevMan 5.3 software for statistical analysis. A fixed-effects model will be used, and data will be synthesized if low heterogeneity exists among included studies (*I*^*2*^ ≤ 50%). We will carry out meta-analysis if there are more than 2 studies on the same interventions, and outcome measurements. On the other hand, a random-effect model will be utilized, and subgroup analysis will be conducted if there is significant heterogeneity among included studies (*I*^*2*^ > 50%). In addition, meta-regression test will be performed.

#### 2.5.8 Publication bias

We will apply Funnel plot and Egger regression test to identify publication bias if there are more than 10 eligible studies.^[25]^

#### 2.5.9 Subgroup analysis

We will investigate the source of heterogeneity using subgroup analysis based on different interventions, controls, and outcomes.

#### 2.5.10 Sensitivity analysis

We will conduct sensitivity analysis to examine the robustness and satiability of outcome results by removing studies with high risk of bias.

## 3 Discussion

Previous studies have reported that ulinastatin is efficacious in the treatment of patients with SAP. However, its conclusion is still inconsistent. Thus, this study will provide a detailed summary of the efficacy and safety of ulinastatin compared to other comparators in the treatment of SAP based on the present evidence, and to assess the clinical efficacy of ulinastatin. The results of this study will provide comprehensively assessment on the efficacy and safety of ulinastatin for treating patients with SAP, and will provide helpful evidence for the clinical practice and future studies.

## Author contributions

**Conceptualization:** Jian-hui Yao, Wei-min Li.

**Data curation:** Jian-hui Yao, Wei-min Li.

**Formal analysis:** Jian-hui Yao.

**Investigation:** Wei-min Li.

**Methodology:** Jian-hui Yao.

**Project administration:** Wei-min Li.

**Resources:** Jian-hui Yao.

**Software:** Jian-hui Yao.

**Supervision:** Wei-min Li.

**Validation:** Jian-hui Yao, Wei-min Li.

**Visualization:** Jian-hui Yao, Wei-min Li.

**Writing – original draft:** Jian-hui Yao, Wei-min Li.

**Writing – review & editing:** Jian-hui Yao, Wei-min Li.
